# All-Trans Retinoic Acid-Induced Pseudotumor Cerebri during Induction Therapy for Acute Promyelocytic Leukemia: A Case Report and Literature Review

**DOI:** 10.1155/2012/313057

**Published:** 2012-06-03

**Authors:** Dylan Holmes, Prakash Vishnu, Russell K. Dorer, David M. Aboulafia

**Affiliations:** ^1^Floyd & Delores Jones Cancer Institute, Virginia Mason Medical Center, Seattle, WA 98101, USA; ^2^Department of Pathology, Virginia Mason Medical Center, 1100 Seattle, WA 98101, USA; ^3^Division of Hematology, University of Washington, Seattle, WA 98195, USA

## Abstract

All-trans retinoic acid (ATRA), a derivative of vitamin A, is an essential component in the treatment of acute promyelocytic leukemia (APL). Though considered to be a relatively safe drug, use of ATRA can lead to several side effects such as retinoic acid syndrome and pseudotumor cerebri (PC). PC is a rare disorder characterized by neurologic and ocular signs and symptoms of increased intracranial pressure, but with normal cerebrospinal fluid composition and normal brain imaging. Most of the previous studies suggest that PC, as a complication of ATRA therapy, occurs predominantly in the pediatric age group. Herein, we report a rare case of ATRA-induced PC in a 38-year-old woman undergoing induction treatment for APL. Symptoms improved with discontinuation of ATRA and treatment with acetazolamide. Concomitant administration of medications such as triazole antifungals which influence the cytochrome P-450 system can exacerbate this potential complication of ATRA. In this paper, we also review the current literature, provide a descriptive analysis of clinical features, and discuss the principles of management of ATRA-induced PC.

## 1. Introduction

Acute promyelocytic leukemia (APL) is a distinct clinicopathologic disorder that accounts for 10% to 15% of cases of acute myeloid leukemia (AML). The unique features of APL have been well chronicled [1, 2] and include a characteristic morphologic appearance [3]; a reciprocal translocation between the long arm of chromosomes 15 and 17, leading to fusion of promyelocytic leukemia (PML)-promoter gene to the retinoic acid receptor (RAR)-*α* gene, which results in the formation of *PML-RAR-*α** fusion gene, younger age of onset, and a severe coagulopathy with a high incidence of early fatal hemorrhage [1, 2, 4–7]. White blood count (WBC) at presentation has been identified as the single most important prognostic factor for clinical outcome [8, 9]. 

All-trans retinoic acid (ATRA), a derivative of vitamin A, when combined with anthracycline-based chemotherapy yields a complete remission (CR) rate in excess of 90% in clinical trials involving patients with APL [9–11]. However, ATRA has also been associated with several side effects, including skin problems (dryness, peeling, itching, and sun sensitivity), reversible elevation in liver enzymes, abnormal lipid levels, hypothyroidism, and headaches. Less commonly, ATRA has been associated with cerebral and myocardial infarction, corneal deposits secondary to hypercalcemia, scrotal ulcerations, Sweet's syndrome, Fournier's gangrene, APL differentiation syndrome, and pseudotumor cerebri (PC) [5, 7, 9, 12–14]. PC is characterized by symptoms and signs of increased intracranial pressure, including headache, diplopia, and papilledema, with a normal cerebrospinal fluid (CSF) composition, and brain imaging findings [15–17]. (Table 1) Papilledema, though a common manifestation of PC, is not an essential criterion for its diagnosis [16, 18]. The exact pathogenesis of ATRA-induced PC has not been established [19, 20]. Several previous studies report, that PC is a complication of ATRA therapy occurring predominantly in pediatric patients usually within 2 weeks of initiation of treatment [19]. 

Herein, we describe a case of ATRA-induced PC in a middle-aged woman which occurred while she was recovering from ATRA-based induction chemotherapy treatment for APL. We also review the current literature and discuss the managing principles of ATRA-induced PC.

## 2. Case Report

**Figure 1:**
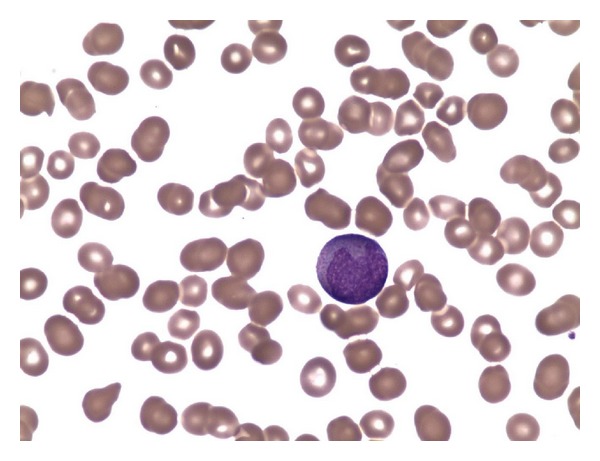
*Peripheral blood film showing promyelocytes. *The peripheral blood film revealed frequent promyelocytes, most of them with multiple Auer rods. Flow cytometric analysis confirmed that these cells were immature promyelocytes expressing CD45 (dim) CD33 (dim), and CD13 and negative for CD34, HLADR, CD14, CD15, CD64, and CD16. Additional FISH studies confirmed the presence of t(15; 17) PML-RARA fusion seen in acute promyelocytic leukemia.

A previously healthy, but obese 38-year-old Native American female, sought surgical evaluation for a left ulnar nerve entrapment syndrome. A routine preoperative laboratory assessment demonstrated a hematocrit of 30%, WBC of 3 × 10^9^/L (48% lymphocytes, 6% monocytes, 16% segmented polymorphonuclear cells, 25% promyelocytes, and 2% blasts) and a platelet count of 13 × 10^9^/L. Further blood test including a coagulation panel showed normal electrolytes and hepatic transaminases, mildly elevated serum lactate dehydrogenase at 298 U/L, prothrombin time of 14.5 seconds (normal 11–13.5), partial thromboplastin time of 35 seconds (normal 25–34), fibrinogen of 762 mg/dL (normal, 212–470), thrombin time of 13 seconds (normal, 15–19), and D-dimer 9.64 *μ*g/mL (normal, <0.40). Peripheral blood film showed several circulating blasts with coarse reddish-purple granules and Auer rods in the cytoplasm, convoluted nuclei, prominent nucleoli and fine open chromatin consistent with promyelocytes. Bone marrow biopsy and aspirate revealed a hypercellular marrow dominated by sheets of promyelocytic-appearing blasts. (Figure 1) Immunohistochemical studies showed that the blasts were positive for CD117 and myeloperoxidase, stained dimly for CD45, and did not express HLA-DR. Cytogenetic analysis demonstrated the characteristic t(15; 17) translocation and FISH analysis confirmed the presence of a *PML/RARA *rearrangement. She began induction chemotherapy (idarubicin 12 mg/m^2^/day IV, days 1–3 and cytosine arabinoside [Ara-C] 100 mg/m^2^/day IV, days 1–7) in conjunction with ATRA (45 mg/m^2^ daily in two divided doses PO). On day 7, she was prescribed fluconazole 400 mg daily and levofloxacin 500 mg daily for antifungal and antibacterial prophylaxis, respectively. On day 17, she reported a throbbing and persistent frontal headache which was accompanied by photosensitivity, nausea, and vomiting. Her neurologic exam was unremarkable. Ophthalmologic exam showed bilateral papilledema but no retinal hemorrhages. A lumbar puncture showed a CSF opening pressure of 300 mm of water (normal <200) with normal biochemical and cytologic findings. Magnetic resonance imaging of the brain was normal. ATRA was withheld and she received 1,000 mg of acetazolamide twice daily in conjunction with standard antiemetics and analgesics. Over the course of the next week, her neurologic and ocular symptoms improved, but they did not completely resolve until 2 days after fluconazole was also discontinued. She has since completed consolidation chemotherapy consisting of two cycles of arsenic trioxide (0.15 mg/kg/day, IV, 5 days/week for 5 weeks), followed by two cycles of ATRA (45 mg/m^2^/day in two divided doses, PO, days 1–7) and daunorubicin (50 mg/m^2^ IV, days 1–3) without recrudescent neurologic symptoms [21]. She then completed ATRA maintenance every other week for a year in conjunction with daily 6-mercaptopurine and weekly methotrexate [22]. She remains in clinical and molecular CR at 18 months of followup.

## 3. Discussion

PC is a rare disorder with an annual incidence of approximately 1 case per 100,000 people, but predominantly affects obese women of childbearing age [23]. It manifests with headache, nausea, and vomiting, as well as pulsatile tinnitus and diplopia. If untreated, it can cause swelling of the optic disc, which may lead to progressive optic atrophy and blindness [24]. Though the exact etiology of PC is not clear, several theories have been proposed such as increased production of CSF, increased blood flow to brain tissue, and increased venous outflow resistance [25].Several medical conditions such as obstructive sleep apnea, pregnancy, Behcet's disease, and thyroid dysfunction have been implicated as risk factors for PC. Also, a number of medications have been casually associated with PC, including oral contraceptives, various antibiotics, thyroid replacement, corticosteroid withdrawal, and lithium [26–32]. Additionally, a strong link has been identified with administration of growth hormones, tetracycline and related compounds and vitamin A derivatives, including ATRA [15, 20, 33–36]. 

It is unclear how ATRA causes PC. One hypothesis suggests that retinoids enhance production of CSF and also alter the lipid constituents of choroid plexus and arachnoid villi, disrupting the normal transport systems and impeding the absorption of CSF [37]. The maximum tolerated ATRA dose in adults is 150 mg/m2/day, and 45–60 mg/m^2^/day for children, but PC has occurred at much lower doses in both groups [13, 14, 38]. ATRA is oxidized by the cytochrome P-450 system including isoforms CYP2C8, CYP2C9, and CYP3A4. Concomitant administration of drugs that inhibit or are metabolized by this system, most notably triazole antifungals, can lead to toxic ATRA concentrations [39, 40]. 

To identify further cases of ATRA-induced PC, we performed a systematic review of peer-reviewed publications using Medical Subject Headings (MeSH), PubMed/Medline, and Google Scholar databases. Keywords were used alone and with the modifiers of “*acute promyelocytic leukemia*,” “*pseudotumor cerebri*,” and “*tretinoin*” or “*all-trans retinoic acid*.” We also examined the bibliographies of each relevant article for additional references. Only publications in English were incorporated in our review. Including our index patient, we identified 21 case reports of PC occurring in patients who received ATRA as a component of APL treatment [14, 19, 20, 38, 40–56]. We identified another 20 reports of ATRA-induced PC in larger scale studies. The cohort size of these studies ranged from 9 to 576 (median: 26) and included a total of 763 patients [13, 55, 57–62]. Nevertheless, adequate information regarding the patients' clinical presentation and outcome was available in only two instances [13, 62]. We, therefore, accumulated and analyzed data from 23 cases (Table 2). 

The median age at diagnosis of PC was 27 years for females (range: 6 to 38 years) and 16 years for males (range: 4 to 43 years) with a slightly higher preponderance of incidence among females (female to male ratio of 1.3 : 1). Data about the role of body mass index (BMI), in ATRA-induced PC was not readily available; only three case reports provided information regarding patients' BMI, which were reportedly normal. As defined by the Centers for Disease Control and Prevention, our patient was obese (BMI 32) [63]. 

Neurologic symptoms were reported in 22 of the patients, of whom all complained of headache, 11 (50%) had diplopia (often with cranial nerve VI palsy), and 7 (32%) had nausea and vomiting. Less common complaints that led to a diagnosis of PC were blurred or distorted vision (23%), photosensitivity (9%), tinnitus (5%), and convergent strabismus (5%). Presenting signs of PC were described in 21 cases and included 5 (24%) with visual field changes such as blind spot enlargement and decreased visual acuity. Papilledema was a uniform finding in the 21 cases in which retinal exams were described.

The median time to the diagnosis of PC after beginning of ATRA therapy was 14 days. (Range: 7 days to 10 months) (Table 2) PC most often occurred during induction therapy (*n* = 18, 78%) but also occurred during consolidation therapy (*n* = 3, 13%) and during maintenance therapy (*n* = 8, 35%).

Strategies for treating PC were provided in all 23 cases. For 17 patients (74%), ATRA was withheld soon after PC was recognized. ATRA was also withheld in three other cases but only after other therapeutic interventions were first implemented. In 7 of the 20 cases (35%) where ATRA was held, PC resolved with no further treatment. In the 13 other cases, signs and symptoms resolved following therapeutic lumbar punctures (5 of 13 cases, 38%) and the use of medications, most notably diuretics—mannitol, glycerin, and acetazolamide (11, 85%); corticosteroids (4, 31%); and/or analgesics (3, 23%). Neurologic symptoms resolved within a median of seven days (range: 1/2 day to 25 weeks) after ATRA was discontinued.

In three cases, ATRA was continued despite the diagnosis of PC. In one instance, the patient was treated with acetazolamide alone. Papilledema resolved within a month, although he continued to have visual complaints at three months followup. In the other two instances, individuals were managed with high-volume therapeutic lumbar punctures; neurologic symptoms and increased cranial pressure resolved within five and seven days, respectively. Including our index patient, a total of eight patients (35%) were rechallenged with ATRA after their neurologic condition improved. In all but our index case, PC symptoms recurred after patients were rechallenged with ATRA, yet in two of these cases PC symptoms were sufficiently mild that no therapeutic intervention was required. CNS symptoms resolved in three of the other five cases following acetazolamide use. ATRA withdrawal and subsequent reintroduction at a reduced dose and prophylactic administration of acetazolamide prior to a subsequent ATRA rechallenge are additional strategies that clinicians have used in the context of PC treatment.

PC through inhibition of CYP enzymes and potentiation of ATRA by triazole antifungals has been described previously in only two instances [14, 56]. In retrospect, our patient had multiple risk factors for PC. She was an obese premenopausal female who received ATRA for treatment of APL. In addition, she received highdoses of fluconazole for antifungal prophylaxis while she was receiving ATRA. This may have played an important role in promoting PC by increasing ATRA drug levels (Table 3). Of note, her symptoms improved partially after we withheld ATRA but did not resolve completely until 48 hours after fluconazole was also discontinued.

## 4. Conclusion

In summary, PC is a well-described syndrome which classically has been associated with obese women of childbearing age. Although the pathogenesis is not well understood, it has also been frequently reported in the context of ATRA treatment for APL. In our literature survey, treatment of PC most often included suspension of ATRA until neurologic symptoms abated. For patients with PC whose neurologic complaints do not improve after withholding ATRA, use of acetazolamide, therapeutic high-volume lumbar punctures, dexamethasone, and analgesics may be useful. Patients may be rechallenged with ATRA once neurologic complaints improve, but symptoms recur in most cases. Since ATRA is an important component of all phases of APL treatment, use of prophylactic acetazolamide prior to a subsequent ATRA rechallenge may mitigate risk of recrudescent PC. Drug interactions, including the one we highlight in this case report between triazole antifungals such as fluconazole and ATRA, must also be kept in mind because fluconazole is a commonly used antifungal agent which is often employed during treatment of acute leukemia.

## Figures and Tables

**Table 1:** Modified Dandy criteria for diagnosis of pseudotumor cerebri [16].

(1) If symptoms and signs are present, they may only reflect those of generalized intracranial hypertension or papilledema.	
The most common symptoms reflecting generalized intracranial hypertension are headache, pulsatile intracranial noises, and double vision. Symptoms reflecting papilledema include transient visual obscurations and peripheral visual loss.	

(2) Elevated intracranial pressure must be documented with the patient lying in the lateral decubitus position.	
A lumbar CSF opening pressure greater than 250 mm H2O is indicative of this disorder. Readings between 200 mm and 250 mm H2O are nondiagnostic.	

(3) CSF must be normal.	
There must be no evidence of pleocytosis, cellular atypia, or hypoglycorrhachia, and CSF protein levels should be normal.	

(4) There must be no evidence of hydrocephalus, mass, structural, or vascular lesion on MRI or contrast-enhanced CT for typical patients and on an MRI and MR venography for atypical patients.	
VST is rare in typical patients (i.e., an obese woman of childbearing age). Therefore, a CT is sufficient even though it cannot detect VST. The incidence of VST and other vascular lesions increases significantly in atypical patients. Consequently, an MRI or MR venography scan is warranted given their heightened ability at detecting these differential disorders.	

(5) No other cause of pseudotumor cerebri can be identified.	
CSF; cerebral spinal fluid; MRI; magnetic resonance imaging; CT; computerized tomogram; and VST; venous sinus thrombosis.	

**Table 2:** Case reports of ATRA-induced pseudotumor cerebri during ATRA treatment for APL.

Case	Age/sex	ATRA dosage (mg/m^2^/day)	ATRA treatment duration prior to PC occurrence [treatment cycle]	PC presentation	PC treatment	PC resolution (days)	Long-term follow up/comments
Index case	38/female	45 mg/m^2^/day	17 days [induction]	Headache, papilledema, 300 mm H_2_O CSF pressure, nausea, vomiting, and photosensitivity	Acetazolamide, antiemetics, and analgesics; withdrawal of ATRA and fluconazole	5 days after withdrawal of ATRA and 1 day after withdrawal of fluconazole	Successful ATRA rechallenge; CR at 12+ months

Jeddi et al. [13]	35/female	45 mg/m^2^/day	25 days [induction]	Headache, papilledema, 500 mm H_2_O CSF pressure, diplopia, and strabismus	Corticosteroids, repeated lumbar punctures; withdrawal of ATRA	2 days after withdrawal of ATRA	Participant in Spanish PETHEM LPA99 trial

Vanier et al. [14]	4/male	*1st course: *45 mg/m^2^/day *2nd course:* 33.75 mg/m^2^/day *3rd course: *Initially 13.5 mg/m^2^/day, increased to 45 mg/m^2^/day	21 days−1 day after beginning 100 mg/day fluconazole [induction]	Headache, papilledema, >200 mm H_2_O CSF pressure, and vomiting	*1st course: *Withdrawal of ATRA *2nd course:* ATRA dose reduction followed by withdrawal *3rd course: *Further ATRA dose reduction and withdrawal of fluconazole	*1st course:* 1 day after withdrawal of ATRA *2nd course:* N/A *3rd course:* 1 day after withdrawal of fluconazole (ATRA maintained)	Multiple ATRA rechallenges: unsuccessful 1st rechallenge at 75% of therapeutic dose, successful 2nd rechallenge at 30% of therapeutic dose, tolerated increase to therapeutic dose after fluconazole withdrawal

Visani et al. [19]	16/male	45 mg/m^2^/day	31 days [induction]	Headache, papilledema, diplopia, tinnitus, and visual field changes	Acetazolamide; withdrawal of ATRA, therapeutic lumbar puncture	15 days after withdrawal of ATRA	Acetazolamide use ineffective but subsequent lumbar puncture successfully relieved PC signs/symptoms; CR at 17+ months

Yeh et al. [20]	27/female	45 mg/m^2^/day	23 weeks [maintenance]	Headache, papilledema, 230 mm H_2_O CSF pressure, and blurred vision	Withdrawal of ATRA	4 weeks after withdrawal of ATRA	N/A

Schroeter et al. [38]	8/female	25 mg/m^2^/day	65 days [consolidation]	Headache, papilledema, diplopia, nausea, vomiting, and left cranial nerve IV palsy	Corticosteroids, mannitol, acetazolamide, and analgesics; withdrawal of ATRA	1 week after withdrawal of ATRA	CR at day 90+

Guirgis MF and Lueder GT [40]	Case A: 16/female	*1st course: *70 mg/day *2nd course: *N/A	*1st course: *1 week [induction] *2nd course: *4 weeks [induction]	*1st course:* headache, papilledema, and visual field changes *2nd course: *headache, papilledema, 370 mm H_2_O CSF pressure	*1st course:* lumbar puncture *2nd course:* acetazolamide (250 mg BID) and therapeutic lumbar puncture	*1st course: *immediate symptomatic relief after lumbar puncture *2nd course: *5 months after administration of acetazolamide and 6 months 9 days after 1st withdrawal of ATRA	Multiple ATRA rechallenges with symptom recurrence during 2nd ATRA course, PC resolution at 5+ months
	Case B: 17/male	90 mg/day	2 weeks [induction]	Headache, papilledema, and visual field changes	Acetazolamide (250 mg TID)	4 weeks after administration of acetazolamide	Maintained ATRA despite signs/symptoms of PC, acetazolamide slowly tapered over two months following PC resolution, persistent blind spots but no papilledema or other symptoms during follow-up

Chen et al. [41]	17/female	45 mg/m^2^/day	8 weeks [maintenance]	Headache, papilledema, 265 mm H_2_O CSF pressure, diplopia, esotropia, and left cranial nerve VI palsy	Withdrawal of ATRA	4 weeks after withdrawal of ATRA	N/A

Selleri et al. [42]	31/female	45 mg/m^2^/day	10 months of continuous ATRA following APL relapses × 2 [maintenance]	Headache, papilledema, 580 mm H_2_O CSF pressure, diplopia, and blurred vision	Withdrawal of ATRA	3 weeks after withdrawal of ATRA	Patient underwent ASCT and in CR at 24 months

Naderi et al. [43]	20/female	40 mg/m^2^/day	13 days [induction]	Headache, papilledema, 390 mm H_2_O CSF pressure, nausea, and vomiting	Therapeutic lumbar punctures	Day 27 of continued ATRA treatment	Maintained ATRA despite signs/symptoms of PC

Tiamkao et al. [44]	35/female	60 mg/day	14 days [induction]	Headache, papilledema, 300 mm H_2_O CSF pressure, blurred vision, and visual field changes	Withdrawal of ATRA	1 week after withdrawal of ATRA	N/A

Mishra et al. [45]	6/female	45 mg/m^2^/day	16 days [induction]	N/A	Withdrawal of ATRA	N/A	Developed Sweet's Syndrome and benign thymic hyperplasia after induction therapy, achieved a CR (timeline N/A)

Sakamoto et al. [46]	11/male	*1st course: *initially 47 mg/m^2^/day, decreased to 39 mg/m^2^/day *2nd course:* 39 mg/m^2^/day	*1st course:* 10 days [induction] *2nd course: *2 days [maintenance]	Headache, nausea	*1st course: *glycerol; ATRA dose reduction followed by ATRA withdrawal *2nd course: *biphosphonate for hypercalcemia	*1st course:* 1 week after withdrawal of ATRA *2nd course: *N/A	Glycerol and ATRA dose reduction ineffective but subsequent ATRA withdrawal relieved PC signs/symptoms; hypercalcemia at day 25, biphosphonate use successfully resolved hypercalcemia and PC; CR at day 46+

Decaudin et al. [47]	16/male	45 mg/m^2^/day	13 days [induction]	Headache, papilledema, 260 mm H_2_O CSF pressure, diplopia, photosensitivity, nuchal stiffness, and bilateral cranial nerve VI paresis	Therapeutic lumbar punctures	3 weeks after withdrawal of ATRA	Maintained ATRA despite signs/symptoms of PC; CR at 12+ months

Sano et al. [48]	18/male	45 mg/m^2^/day	23 days [induction]	Headache, papilledema, 350 mm H_2_O CSF pressure, diplopia, and nausea	Glycerin, withdrawal of ATRA	6 days after withdrawal of ATRA	CR at day 29

Machner et al. [49]	20/female	*1st course: *45 mg/m^2^/day *2nd course: *45 mg/m^2^/day	14 days [induction]	*1st course: *headache, papilledema, 500 mm H_2_O CSF pressure, blurred vision, visual field changes *2nd course: *headache	*1st course*: lumbar puncture, acetazolamide (1000 mg/day) and analgesics, withdrawal of ATRA *2nd course: *continued acetazolamide	1 day after withdrawal of ATRA following 2nd course	Successful ATRA rechallenge caused minimal toxicity (low level stable headache), CR at day 30+, NED and without PC at 3 months

Gallipoli [50]	31/female	45 mg/m^2^/day	17 days [induction]	*1st CT course: *headache, papilledema, 600 mm H_2_O CSF pressure, diplopia, and right cranial nerve VI palsy *2nd–4th CT courses and maintenance therapy: *headache and elevated CSF pressure	*1st CT course: *therapeutic lumbar punctures, dexamethasone, and acetazolamide, withdrawal of ATRA *2nd–4th CT courses and maintenance therapy: *therapeutic lumbar punctures and acetazolamide (1000 mg/day)	*1st CT course:* N/A *2nd–4th CT courses and maintenance therapy: *N/A	Multiple ATRA rechallenges at full dose induced PC during consolidation and maintenance therapy, PC signs/symptoms steadily resolved during all CT courses and maintenance therapy (timeline N/A), CR after induction therapy (timeline N/A)

Varadi et al. [51]	17/female	45 mg/m^2^/day	N/A [maintenance]	Headache, papilledema, 340 mm H_2_O CSF pressure, diplopia, blurred vision, and right cranial nerve VI palsy	Dexamethasone and acetazolamide, withdrawal of ATRA, allogeneic BMT	N/A	1st ATRA course induced hyperleukocytosis, ATRA rechallenge induced APL relapse and PC symptoms; allogeneic BMT from HLA-identical brother, achieved a CR (timeline N/A), NED 27 months post-BMT

Naithani et al. [52]	9/male	*1st course:* 45 mg/m^2^/day *2nd course: *10 mg bid on day 16, escalated to 30 mg bid on day 19 and 45 mg/m^2^/day on day 21 *3rd course:* “low dose” on day 26, escalated to 30 mg bid	*1st course: *12 days [induction] *2nd course:* 7 days [induction] *3rd course: *N/A (no PC)	*1st course:* headache, papilledema, diplopia, and vomiting *2nd course:* headache, vomiting *3rd course: * N/A (no PC)	*1st course: *Acetazolamide and mannitol, withdrawal of ATRA *2nd course: *Withdrawal of ATRA *3rd course: *N/A (no PC)	1/2 day after withdrawal of ATRA following 2nd course	Multiple ATRA rechallenges with successful 2nd rechallenge, CR at 5 weeks

Colucciello [53]	30/male	N/A	14 days [induction]	Headache, papilledema, 225 mm H_2_O CSF pressure, diplopia, and cranial nerve VI palsy	Withdrawal of ATRA	6 weeks after withdrawal of ATRA	Achieved a CR with multiagent CT (timeline N/A)

Ganguly [54]	43/male	45 mg/m^2^/day	*1st course: * 9 days [induction] *2nd course:* 7 days [maintenance]	Headache, papilledema	*1st course:* acetazolamide (500 mg/day) *2nd course:* Withdrawal of ATRA	N/A	Multiple ATRA rechallenges during maintenance therapy, 2nd rechallenge successful with prophylactic acetazolamide (500 mg/day)

Smith et al. [62]	6/male	Initially 45 mg/m2/day, escalated to 80 mg/m2/day	7–10 days [induction]	Headache, papilledema, and elevated opening CSF pressure	Withdrawal of ATRA	N/A	Opening CSF pressure elevated but not measured, successful ATRA rechallenge (60 mg/m2/day) led to 5 month ATRA course following neurologic symptom resolution

CSF: cerebral spinal fluid; ATRA: all-trans retinoic acid; CR: complete remission; PC: pseudotumor cerebri; N/A: not available; ASCT: autologous stem cell transplant; CT: chemotherapy; BMT: bone marrow transplantation; NED: no evidence of disease.

**Table 3:** Common drug interactions with tretinoin [39].

Drug(s)	Interaction
Antifibrinolytic agents (e.g., aminocaproic acid, aprotinin, and tranexamic acid)	May increase risk of thrombosis during tretinoin therapy.
Drugs that induce cytochrome P-450 system (e.g., barbiturates, carbamazepine, phenytoin, primidone, rifabutin, and rifampin)	May decrease tretinoin concentrations and antineoplastic efficacy.
Drugs that inhibit cytochrome P-450 (e.g., cimetidine, erythromycin, fluconazole, and ketoconazole)	May increase tretinoin concentrations and toxicity. Adjust tretinoin concentrations when necessary.
Drugs that inhibit CYP-2C8 (e.g., atazanavir, gemfibrozil, and ritonavir)	May increase tretinoin concentrations and toxicity. Adjust tretinoin concentrations when necessary.
Estrogen-progestin oral contraceptives	Tretinoin may decrease contraceptive efficacy. The use of at least 1 other effective form of contraception during tretinoin therapy is recommended.
Progestin-only oral contraceptives	Tretinoin may decrease contraceptive efficacy. The use of at least 2 other effective forms of contraception during tretinoin therapy is recommended.
Tetracycline antibiotics (e.g., doxycycline, minocycline, and tetracycline)	May increase risk of PC.
Ethanol	May increase CNS depression. Avoid concomitant use.
Hydroxyurea	Synergistic antineoplastic effects; may increase risk of cell lysis and potentially fatal bone marrow necrosis.
St. John's wort	May decrease tretinoin concentrations and antineoplastic efficacy. Avoid concomitant use.
Vitamin A	Increased risk of vitamin A toxicity. Avoid combination.
